# Lipid lowering efficacy and safety of Ezetimibe combined with rosuvastatin compared with titrating rosuvastatin monotherapy in HIV-positive patients

**DOI:** 10.1186/s12944-015-0054-x

**Published:** 2015-06-19

**Authors:** Ramesh Saeedi, Kevin Johns, Jiri Frohlich, Matthew T. Bennett, Gregory Bondy

**Affiliations:** Department of Pathology & Laboratory Medicine, University of British Columbia, Vancouver, Canada; Experimental Medicine Program, Faculty of Medicine, University of British Columbia, Vancouver, Canada; Division of Cardiology, Department of Medicine, University of British Columbia, Vancouver, Canada; Departments of Medicine and Pathology, Faculty of Medicine, University of British Columbia, Vancouver, Canada

**Keywords:** HIV, Rosuvastatin, Ezetimibe, Lipid profile

## Abstract

**Background:**

HIV-infected patients on antiretroviral therapy frequently develop dyslipidemias and, despite therapy with potent lipid-lowering agents, a high percentage does not achieve guideline recommended lipid targets. In this study, we examined the efficacy of combination treatment with a statin and the cholesterol transport blocker, ezetimibe, *vs.* monotherapy with a statin in HIV-infected patients not achieving lipid goals.

**Methods:**

This was a 12-week, prospective, randomized, open-label clinical trial. Patients were eligible if they had an apolipoprotein B (apoB) >0.80 g/L despite therapy with rosuvastatin 10 mg daily for a minimum of 12 weeks. Patients were randomized to take ezetimibe 10 mg/rosuvastatin 10 mg or rosuvastatin 20 mg for 12 weeks. Percentage and absolute change in apoB (primary outcome), low-density lipoprotein cholesterol (LDL-C), total cholesterol (TC), triglyceride (TG), high-density lipoprotein cholesterol (HDL-C), non-HDL-C, apoliporpotein A1 (apoA1), apoB/apoA1, TC/HDL-C, atherogenic index of plasma (API), and high-sensitivity C-reactive protein (hsCRP) were compared. Changes in safety parameters (such as AST, ALT, CK) and clinical symptoms were also assessed.

**Results:**

Forty-three patients (23 on ezetimibe 10 mg/rosuvastatin 10 mg and 20 on rosuvastatin 20 mg) completed the trial. Baseline characteristics did not differ between the groups. Significant improvements in apoB were seen with both ezetimibe plus rosuvastatin (mean of -0.17 g/L, p < 0.001) and rosuvastatin 20 mg (mean of -0.13 g/L, *p* = 0.03) treatment groups, but did not differ between groups (*p* = 0.53). Significant between-group differences were observed for mean TC (-1.01 mmol/L vs. -0.50 mmol/L, *p* = 0.03), TG (-0.62 mmol/L vs -0.17 mmol/L, *p* = 0.03), and non-HDL-C (-0.97 mmol/L vs. -0.53 mmol/L, *p* = 0.03) all in favour of the ezetimibe plus rosuvastatin group. Two patients, both in the rosuvastatin 20 mg group, experienced mild myalgias; neither discontinued the study.

**Conclusions:**

The addition of ezetimibe to rosuvastatin appears to be safe in patients with HIV. Furthermore, the combination of ezetimibe and rosuvastatin improved TG, AIP and non-HDL cholesterol levels more than a dose increase in rosuvastatin in patients with HIV-associated dyslipidemia.

## Background

Human immunodeficiency virus (HIV)-associated dyslipidemia is becoming more common with the extensive use of antiretroviral therapy (ART) resulting in an increase in incidence of atherosclerotic cardiovascular disease (ASCVD) [1–6].

Statins have been widely used for prevention of ASCVD [7]. Increasing numbers of individuals with HIV-associated dyslipidemia are receiving statins for secondary prevention of ASCVD [8]; many HIV-infected patients receiving statin therapy do not achieve recommended treatment goals [9]. Furthermore, treatment of HIV-associated dyslipidemia is further complicated by altered statin metabolism and drug interactions with antiretroviral medications both of which increase the risk of statin induced myopathy [9]. Few studies have examined the effect of alternative lipid-lowering agents in HIV-infected patients.

Ezetimibe, an inhibitor of intestinal cholesterol absorption, has been used as a second-line therapy for patients who are unable to tolerate high dose statins or in those who do not achieve therapeutic lipid target levels [10]. In HIV-dyslipidemia, ezetimibe is safe and can be used in patients with poor response to statins and those receiving protease inhibitors [10–14].

Rosuvastatin is a relatively new statin, which is not metabolised by the cytochrome P 3A4 (CYP3A4) enzyme system. As CYP3A4 is often enzyme-inhibited by certain ART therapies, rosuvastatin is preferred in the treatment of ART-associated dyslipidemia. A limited number of trials have assessed the efficacy of rosuvastatin in patients with HIV with all reporting a favorable decline in some of lipid parameters [8, 15–18]. The aim of the current study was to assess whether HIV-infected patients treated with a combination of rosuvastatin 10 mg plus ezetimibe 10 mg show a greater improvement in their lipid profile compared to an increased dose of rosuvastatin (from 10 to 20 mg). Several studies in non-HIV populations have shown that co-administration of rosuvastatin plus ezetimibe is safe, and achieved significant improvements in lipid profiles in high-risk patients compared to monotherapy [10].

## Methods

This prospective, randomized, open-label study was carried out in HIV-infected patients seen at the Immunodeficiency Clinic (IDC)-HIV metabolic Clinic at St. Paul’s Hospital, Vancouver, BC, Canada. We compared the efficacy and safety of co-administration of 10 mg rosuvastatin and 10 mg of ezetimibe versus 20 mg of rosuvastatin over a 15 months period. The protocol and informed consent were approved by the institutional Ethics Board (University of British Columbia).

Patients included in this study were men and women age ≥ 19 years with apolipoprotein B (apoB) >0.8 g/L, maintained on stable dose of rosuvastatin 10 mg for the past 12 weeks and on a stable background of ART for the 12 weeks prior to study. Patients continued the same ART for the subsequent 12 weeks of study.

Main exclusion criteria included serum aspartate aminotransferase (AST) or alanine aminotransferase (ALT) elevations ≥3-fold upper limit of normal (ULN), serum creatine kinase (CK) concentration elevation ≥10-fold ULN, previous adverse reaction to ezetimibe, recent use of ezetimibe (within 30 days of starting the study), a history of significant myopathy or rhabdomyolysis with any statin, current use of gemfibrozil (Lopid), niacin/nicotinic acid, colestipol (Colestid), cholestyramine (Novo-Cholamine), or any agent with a potential drug-drug interaction, pregnant or breastfeeding woman or expecting to conceive or donate eggs during the study; males planning to impregnate a woman or provide sperm donation during the study; use of excessive (more than one drink a day for a woman and two drinks for a man) amounts of alcohol or recreational drugs; or previous or current liver disease.

Patients were randomized in a 1:1 ratio to either an increased daily dose of rosuvastatin to 20 mg, or to 10 mg ezetimibe added on to their current 10 mg rosuvastatin daily dose. Serum samples were obtained and analyzed at the baseline and after 12 weeks of treatment for lipid profile, safety and inflammatory parameters. Also, at the end of the study (12 weeks) participants were asked questions regarding side effects (such as myalgia and gastrointestinal side effects).

The primary outcome was the percent change in serum apoB, between participants treated with rosuvastatin 20 mg versus participants treated with ezetimibe 10 mg plus rosuvastatin 10 mg. Even though, calculated or measured LDL-C concentration has been known as the major goal of lipid lowering therapies, many individuals may experience progression of atherosclerosis or cardiovascular events despite having ideal LDL-C concentrations. Barter et al have shown that predicting the future CVD is best reflected by apoB concentration [19]. ApoB, which measures numbers of atherogenic particles in plasma, is superior to LDL-C not only as an index of cardiovascular risk but also as a guide to the adequacy of lipid lowering therapy. Secondary outcomes included the percent and absolute change in serum concentrations of low-density lipoprotein cholesterol (LDL-C), total cholesterol (TC), triglycerides (TG), high-density lipoprotein cholesterol (HDL-C), TC/ HDL, non- HDL-C, apolipoprotein A1 (apoAI), apoB/apoAI, atherogenic index of plasma (AIP), fasting blood glucose (FBG), and high-sensitivity C-reactive protein (hsCRP) between participants treated with rosuvastatin alone versus participants treated with rosuvastatin and ezetimibe. Further secondary endpoints included within group changes in the above endpoints and the assessment of safety parameters, specifically incidence of complications as measured by an increase in AST and/or ALT ≥3-fold ULN and a CK ≥10-fold ULN in participants treated with rosuvastatin 20 mg versus participants treated with ezetimibe 10 mg plus rosuvastatin10 mg.

For the primary outcome, with a sample size of 50 patients planned for randomization, the study was anticipated to have at least 90 % power to demonstrate a difference between ezetimibe co-administrated with rosuvastatin and the comparative increased dose rosuvastatin monotherapy, assuming a reduction of apoB of 0.25 g/L, a SD of 0.27 g/L (the type one error of 0.05 and type two error of 0.1).

### Statistical analysis

Data are expressed as mean ± SD. Assumptions of normality were tested by Shapiro-Wilks tests and review of plots. The primary analysis was to compare treatment effects between groups. For variables with a symmetrical distribution, analysis of covariance (ANCOVA) adjusted for baseline values were used. For variables with skewed distributions, between-treatment changes from baseline were compared with Mann-Whitney U tests. The secondary analysis was to compare changes within each group with paired t-tests or Wilcoxon rank-sum tests, as appropriate. Calculations were performed using computer software (SPSS/Mac v. 19.0.0).

Any patients who did not return for the 12-week visit were excluded from the analysis except those who had withdrawn for reason of intolerance or toxicity. These patients were considered not to have changed from baseline if they did not appear for their follow-up visit. The proportion of patients who have experienced an increase in AST and ALT ≥3-fold ULN or a CK ≥10-fold ULN were compared between treatment groups using the Fisher’s exact test.

## Results

### Patient demographics and baseline characteristics

Due to slow enrollment accrual only 46 patients were randomized and 43 patients (23 rosuvastatin plus ezetimibe, 20 rosuvastatin) completed the 12 week study. Three patients out of 46 patients were excluded, 2 due to elevated liver enzymes and one due to complaints of muscle soreness. Baseline characteristics of the study population are summarized in Table 1. There were no differences between groups in terms of age or clinical characteristics of HIV. However, despite randomization, there were some observable differences between groups in terms of cardiovascular risk factors. More patients receiving ezetimibe had a history of hypertension and more were current smokers whereas more patients in the rosuvastatin group self-identified as past smokers (quit within the last 12 months). Baseline lipid and lipoprotein levels were similar between the two groups (Table 2).

**Table 1:** Baseline characteristics of the study subjects*

Characteristic	Ezetimibe add- on group (*n* = 23)	Increased rosuvastatin (*n* = 20)
Age, years†	56.5 ± 7.4	57.0 ± 9.9
Male sex	22 (95.6)	17 (85.0)
Caucasian	19 (82.6)	15 (75.0)
Duration of ARV therapy (months)†	24 (16–34)	25(14-48)
CD4+ cell count (cells/mm3)†	580.0 ± 193	508.0 ± 208
Undetectable VL‡	20 (87.0)	19 (95.0)
PI	21 (91.3)	17 (85.0)
NNRTI	6 (26.1)	7 (35.0)
NRTI	23 (100)	19 (95.0)
Fibrate	4 (17.4)	6 (30.0)
Antihypertensive agents	13 (56.5)	6 (30.0)
Current Smoker	6 (26.1)	1 (5.0)
Previous Smoker§	8 (34.8)	14 (70.0)
Diabetes Mellitus	8 (34.8)	7 (35.0)
Hypertension	13 (56.5)	6 (30.0)
Family History of CVD	10 (43.5)	10 (55.0)
Body Mass Index (kg/m2)†	26.0 ± 4.2	25.3 ± 4.7
Waist Circumference, cm†	98 ± 13.7	93 ± 15.3

*Data are frequency (%) unless otherwise indicated

†Data are mean (±SD)

‡ < 40 copies/mL

§ less than one year of smoking cessation prior to study recruitment

ARV, antiretroviral; VL, viral load; PI, protease inhibitor; NNRTI, non-nucleoside

reverse transcriptase inhibitor; NRTI, nucleoside reverse transcriptase; CVD, cardiovascular disease

**Table 2:** Baseline values for primary and secondary outcomes

	Ezetimibe add- on group (*n* = 23)	Increased rosuvastatin (*n* = 20)
ApoB, g/L	1.06 ± 0.24	1.01 ± 0.38
ApoA1, g/L	1.54 ± 0.27	1.51 ± 0.32
ApoB:ApoA1	0.69 ± 0.21	0.72 ± 0.39
TC, mmol/L	5.23 ± 1.06	5.07 ± 0.50
LDL, mmol/L	2.80 ± 0.93	2.55 ± 0.58
HDL, mmol/L	1.21 ± 0.29	1.29 ± 0.40
TC:HDL	4.92 ± 2.25	4.25 ± 1.15
TG, mmol/L	2.55 ± 1.32	3.05 ± 1.52
AIP	0.29 ± 0.33	0.33 ± 0.33
Non-HDL-C, mmol/L	4.04 ± 1.05	3.78 ± 0.69
Glucose, mmol/L	6.54 ± 1.84	6.16 ± 2.22
hsCRP, mg/L	5.6 ± 10.20	2.8 ± 3.37
Creatinine, μmol/L	91 ± 22.50	98 ± 30.51
ALT, U/L	33 ± 13.80	34 ± 12.70
AST, U/L	29 ± 11.0	30 ± 11.40
CK, U/L	142 ± 100.0	177 ± 85.22

Data are given as mean (±SD)

apoB, apolipoprotein B; apoA1,apolipoprotein A1;TC, total cholesterol; LDL-C, low-density lipoprotein cholesterol; HDL, high-density lipoprotein cholesterol; TG, triglycerides; AIP, atherogenic index of plasma; FBG, fasting blood glucose; hsCRP, high-sensitivity C-reactive protein; AST, aspartate aminotransferase; ALT, alanine aminotransferase; CK, creatine kinase

### Effects of adding ezetimibe to rosuvastatin or increasing rosuvastatin dose on metabolic parameters

The primary and secondary endpoints are displayed in Table 3. Twelve weeks of treatment with either rosuvastatin 20 mg or rosuvastatin 10 mg plus ezetimibe 10 mg significantly decreased TC, LDL, TC/HDL, and apoB. Also, addition of ezetimibe to rosuvastatin resulted in significant improvements in TG, non-HDL-C, apoB/apoAI, and AIP. The improvement in TC and TG was significantly greater with ezetimibe 10 mg plus rosuvastatin 10 mg than with rosuvastatin 20 mg alone.

**Table 3:** Effects of adding ezetimibe to rosuvastatin or increasing rosuvastatin dosage on lipid and metabolic outcomes

	Values after 12 weeks		Change after 12 weeks		*P* Value (Difference Between add on vs. increased dose)
	Ezetimibe add-on group (*n* = 21)	Increased rosuvastatin (*n* = 18)	Ezetimibe add-on group (*n* = 21)	Increased rosuvastatin (*n* = 18)	
ApoB, g/L	0.89 ± 0.25	0.88 ± 0.22	−0.17 ± 0.18*	−0.13 ± 0.25*	0.53
ApoA1, g/L	1.47 ± 0.26	1.56 ± 0.30	−0.07 ± 0.25	−0.05 ± 0.24	0.15
ApoB:ApoA1	0.55 ± 0.29	0.60 ± 0.23	−0.15 ± 0.29*	−0.12 ± 0.33	0.81
TC, mmol/L	4.23 ± 1.03	4.56 ± 0.75	−1.01 ± 0.79*	−0.50 ± 0.63*	0.03
LDL, mmol/L	2.12 ± 0.63	2.07 ± 0.46	−0.68 ± 0.54*	−0.48 ± 0.55*	0.37
HDL, mmol/L	1.21 ± 0.27	1.31 ± 0.48	−0.00 ± 0.29	0.03 ± 015	0.72
TC:HDL	3.29 ± 1.41	3.73 ± 0.92	−1.63 ± 2.86*	−0.52 ± 0.65*	0.09
TG, mmol/L	1.92 ± 0.98	2.88 ± 1.42	−0.62 ± 0.58*	−0.17 ± 0.57	0.03
AIP	0.16 ± 0.30	0.30 ± 0.38	−0.12 ± 0.20*	−0.03 ± 0.12	0.13
Non-HDL-C, mmol/L	3.06 ± 0.98	3.25 ± 0.71	−0.97 ± 0.68*	−0.53 ± 0.13	0.03
FBG, mmol/L	6.87 ± 2.49	5.82 ± 1.29	0.34 ± 1.28	−0.34 ± 1.75	0.15
hsCRP, mg/L	5.19 ± 7.25	1.72 ± 1.87	−0.60 ± 12.5	−1.06 ± 3.48	0.87
Creatinine, μmol/L	98 ± 58	151 ± 265	8 ± 48	53 ± 258	0.43
ALT, U/L	42 ± 22	34 ± 15	9 ± 17*	1.28 ± 16.6*	0.18
AST, U/L	32 ± 21	29 ± 8	4 ± 14	−0.10 ± 8	0.30
CK, U/L	126 ± 63	189 ± 103	−20 ± 90	12 ± 69	0.23

Data are given as mean (±SD)

*Significant change from their baseline (*p* < 0.05)

apoB, apolipoprotein B; apoA1,apolipoprotein A1;TC, total cholesterol; LDL-C, low-density lipoprotein cholesterol; HDL, high-density lipoprotein cholesterol; TG, triglycerides; API, atherogenic index of plasma; FBG, fasting blood glucose; hsCRP, high-sensitivity C-reactive protein; AST, aspartate aminotransferase; ALT, alanine aminotransferase; CK, creatine kinase

Ezetimibe 10 mg plus rosuvastatin 10 mg and rosuvastatin 20 mg monotherapy were generally well tolerated during this 12 week study. Two patients in the rosuvastatin monotherapy group experienced myalgias with one of them experiencing moderate to severe cramping. Neither of these two patients discontinued the study medication. A statistically significant increase in ALT was observed in the ezetimibe plus rosuvastatin group but this increase was small and not likely to be clinically relevant.

Sub-analysis was carried out to determine the proportion of patients reaching primary (LDL-C ≤2.0 mmol/L, 50 % reduction in LDL-C, ApoB ≤0.80 g/L, or non-HDL-C ≤2.6) or secondary (TG <1.7 mmol/L, ApoB:ApoA1 ratio <0.80, hsCRP <2 mg/L) high cardiovascular risk targets [20] as well as the high risk limit for AIP (<0.21) [21]. As illustrated in Table 4, both treatment regimens resulted in an increased proportion of patients reaching primary and secondary targets with the exception of hsCRP, which showed no improvement in the ezetimibe plus rosuvastatin group.

**Table 4:** Primary and secondary targets 12 weeks after treatment with rosuvastatin or rosuvastatin plus ezetimibe

	Primary Targets				Secondary Targets		
	LDL-C ≤2.0 mmol/L	50 % reduction in LDL	ApoB ≤0.80 g/L	non-HDL-C ≤2.6	TG <1.7 mmol/L	ApoB:ApoA1 ratio <0.80	hsCRP <2 mg/L
Rosuvastatin 20 mg	2.07	19 % reduction	0.88	3.25	2.88	0.60	1.72
Rosuvastatin 10 mg + Ezetimibe 10 mg	2.12	24 % reduction	0.89	3.06	1.92	0.55	5.19

LDL-C, low-density lipoprotein cholesterol; apoB, apolipoprotein B;; non-HDL-C, non- high-density lipoprotein cholesterol; TG, triglycerides; apoA1, apolipoprotein A1; hsCRP, high-sensitivity C-reactive protein

## Discussion

The primary findings of this study is that rosuvastatin10 mg/ezetimibe 10 mg combination had a greater effect on lowering non-HDL-C, TG, and AIP as compared to rosuvastatin 20 mg monotherapy. This trial is the first study to evaluate and compare the efficacy and safety of ezetimibe and rosuvastatin 10 mg versus rosuvastatin 20 mg in HIV-infected patients.

This study showed that both ezetimibe 10 mg plus rosuvastatin 10 mg and rosuvastatin 20 mg significantly reduced LDL-C. Addition of ezetimibe 10 mg to rosuvastatin 10 mg had a greater impact on lowering LDL-C than rosuvastatin 10 mg monotherapy (22.5 % reduction vs. 16.5 % reduction, respectively). The magnitude of changes in LDL-C in our study was similar to other studies done in HIV populations where eztimibe was co-administrated with statin therapy; these studies reported a 12-35 % decrease in LDL-C [11–14]. The wide range of changes in LDL-C between studies may be due to other factors such as age of participants, type and dose of statin, type of ART, or other medications. Overall, the percent reduction in LDL-C in our study and studies involving HIV-infected patients is not as high as in studies involving non-HIV infected patients. In non-HIV studies with ezetimibe added on therapy LDL-C was decreased by 25-60 % [10]. This suggests that HIV-infected patients are less affected by this therapy. The 2012 Canadian Cardiovascular Society (CCS) guidelines for the general population have recommended a 50 % reduction in LDL-C from baseline or LDL < 2.0 mmol/L as the target levels for the treatment of moderate and high risk patients [20]. In our center, we treat HIV- positive patients at the moderate risk lipid profile targets. While ezetimibe administration was more effective in reducing LDL-C, neither of the two groups reached therapeutic targets.

The 2012 CCS guidelines also recommend alternative targets including apoB ≤ 0.8 g/L or non-HDL-C ≤ 2.6 mmol/L for the treatment of moderate and high risk patients [20]. Growing evidence indicates that during treatment of dyslipidemia, CVD events correlate more strongly with levels of apoB and non-HDL-C [22]. ApoB has been suggested as an alternative primary treatment target in the 2012 CCS guidelines for individuals with moderate-high CVD [20]. Recently, Piconi et al have suggested that apoB may predict ASCVD in HIV-infected populations [23]. In terms of patients reaching the CCS target of apoB ≤0.80 g/L the results were similar between groups following 12 weeks of treatment. When ezetimibe was added to rosuvastatin, 45 % (from 15.0 % at baseline) of patients reached target compared to 50 % (from 27.8 % at baseline) in patients treated with rosuvastatin alone. These improvements in apoB are similar to those reported in a large trial in non-HIV patients on ezetimibe monotherapy [24]. In addition, only the ezetimibe treated group had a significant reduction in non-HDL-C (24 %) as compared to baseline. In keeping with our findings, others have shown that in HIV-infected patients combination of statin with ezetimibe was more effective in lowering non-HDL-C than statin alone [25]. Borrato et al have shown that treatment of HIV-infected patients with rosuvastatin 10 mg alone for 16 weeks resulted in a significant reduction of LDL-C as well as non-HDL-C with 65 % of them achieving therapeutic targets [26]. In rosuvastatin monotherapy, we did not see any significant changes in non-HDL-C. The differences between their and our study could be due to the type of ART, or other confounding factors. In our study, more patients in the combination group reached TG <1.7 mmol/L (53.3 %) than the increased dose group (15 %).

Another marker for cardiovascular risk that has yet to be thoroughly assessed in the HIV-infected patients is the apoB/apoA1 ratio. Several studies in the non-HIV population have shown that increase in this ratio is associated with an increased risk of CVD [27, 28]. It has been suggested that apoB/apoA 1 ratio can be considered a tool to fine-tune the risk assessment and thus the targets of therapy [5]. This ratio may be valuable in determining the risk or response to treatment in the HIV-infected individuals. Our study showed that both treatments resulted in a reduction in apoB/apoA1, albeit this reduction was statistically significant only in the ezetimibe-treated patients. However, it should be noted that among our study patients, an elevated apoB/apoA1 ratio (>0.80) was not prevalent (38 % of patients). This may be due to the fact that at baseline all these patients were on 10 mg of rosuvastatin. Accordingly, nearly every patient reached the apoB/apoA1 target following 12 weeks of treatment. This could suggest that CVD risk is not greatly elevated in the HIV-infected population but as our sample size is small and further investigation into prevalence of elevated apoB/apoA1 ratio in this population is warranted.

The reported effects of ezetimibe on other lipid parameters and hsCRP have been variable. Our study results showed a significant decrease in TC by 19 % in the ezetimibe plus rosuvastatin group and 10 % in rosuvastatin monotherapy group which is similar to the 10-32 % reduction previously reported [11, 12, 29]. Also, hsCRP was lowered by 38 % in rosuvastatin 20 mg group and 10 % in combination of ezetimibe and rosuvastatin group. In neither group reached statistical significance. The hsCRP is an inflammatory biomarker, levels of which are associated with risk of CVD. The Emerging Risk Factors Collaboration has shown an stepwise increase in risk of CVD for hsCRP levels between 0.5 and 20 mg/L with hsCRP > 2.0 mg/L associated with a hazard ratio of CVD of 1.5 [30]. However, this was attenuated after correction for age, sex, diabetes, TG, and HDL-C. Also, Mendelian randomization studies have demonstrated that hsCRP is not related to risk of CVD and not a target of therapy [31, 32]. For HIV-infected patients, the role of hsCRP may be less clear because the results could be confounded by other factors such as other comorbidities and risk factors [5]. We did not see any significant changes in HDL-C in either group which is consistent with some studies [12, 14].

Here we showed that the combination of ezetimibe and rosuvastatin decreased TG (0.62 mmol/L, 24.0 %) and AIP (0.12, 42.6 %) whereas no such improvement was seen in the rosuvastatin 20 mg group following 12 weeks of treatment. A novel and not fully investigated marker of CVD risk in the HIV-infected population is AIP. The AIP, calculated as logarithm (TG/HDL-C), correlates with size of LDL-C particles, rate of esterification of plasma cholesterol independent of apoB concentration [33]. It is used as a predictive marker for atherogenic risk and cardiovascular risk and is a useful measure of response to pharmacological intervention [33, 34]. Thus, AIP may prove to be a useful predictor of risk in this population given its predictive utility in non-HIV studies [20] and the fact that it combines two parameters that are often adversely affected by HIV and ART. At baseline, 60 % of our study cohort were above the high-risk AIP target of >0.21 [21]. This was significantly improved in the combination therapy group. The 20 mg rosuvastatin monotherapy improved AIP by 8 % but this did not reach statistical significant.

Addition of ezetimibe to rosuvastatin was found to be well tolerated with an improvement in clinical and biochemical safety profile. As it does not interfere with CYP450 system and there is low probability of drug-drug interaction between ezetimibe and HIV drugs.

### Limitations

There are several limitations to this study. First this was a single center study with a small sample size. A 12 week study duration may be considered short and was not designed to evaluate possible the long-term adverse effects of ezetimibe. Furthermore, it is not clear whether the improved reductions in lipid endpoints with ezetimibe add on therapy will correlate with cardiovascular endpoints when compared to increased statin therapy in this population. In addition, despite randomization, there were differences between the groups, especially with respect to smoking and hypertension.

## Conclusions

We have demonstrated that addition of ezetimibe 10 mg to rosuvastatin 10 mg is a more effective treatment for dyslipidemia in HIV-infected population than increasing dose to 20 mg. Despite lowering of apoB within groups, there was no statistically significant difference in apoB between the treatment groups. Ezetimibe was well-tolerated and no serious adverse events were identified. Trials in HIV-infected populations are required to assess the effectiveness of ezetimibe and/or statins on cardiovascular outcome.
